# The Effects of the Early Start Denver Model for Children with Autism Spectrum Disorder: A Meta-Analysis

**DOI:** 10.3390/brainsci10060368

**Published:** 2020-06-12

**Authors:** Elizabeth A. Fuller, Kelsey Oliver, Sarah F. Vejnoska, Sally J. Rogers

**Affiliations:** Department of Psychiatry and Behavioral Sciences, University of California, Davis MIND Institute, Sacramento, CA 95817, USA; efuller@ucdavis.edu (E.A.F.); kaeoliver@ucdavis.edu (K.O.); sfvejnoska@UCDAVIS.EDU (S.F.V.)

**Keywords:** autism, early intervention, Early Start Denver Model

## Abstract

This meta-analysis examined the effects of the Early Start Denver Model (ESDM) for young children with autism on developmental outcome measures. The 12 included studies reported results from 640 children with autism across 44 unique effect sizes. The aggregated effect size, calculated using a robust variance estimation meta-analysis, was 0.357 (*p* = 0.024), which is a moderate effect size with a statistically significant overall weighted averaged that favored participants who received the ESDM compared to children in control groups, with moderate heterogeneity across studies. This result was largely driven by improvements in cognition (*g* = 0.412) and language (*g* = 0.408). There were no significant effects observed for measures of autism symptomology, adaptive behavior, social communication, or restrictive and repetitive behaviors.

## 1. Introduction

The estimated prevalence of autism spectrum disorders (ASD) has continuously increased in recent decades with the most current prevalence rates estimating that 1 in 54 children under 8 years of age are diagnosed with ASD [1]. This includes an increasing prevalence of young children being diagnosed partly due to the more widespread use of early screening measures and adaptations to diagnostic tools that has led to children being diagnosed with ASD as early as 12–18 months [2]. Children this young need early intervention services that have been designed for and tested with them, given the many developmental and social-emotional differences of infants and toddlers when compared to preschoolers and older children [3]. Given the increasing prevalence estimates of ASD and the high cost of ASD treatments [4], it is critical to identify ASD intervention approaches that are appropriate and effective for supporting young children and their families.

### 1.1. Naturalistic Developmental Behavioral Interventions

Naturalistic developmental behavioral interventions (NDBIs) are one class of ASD interventions that are particularly geared towards the needs of young children [5]. The term NDBI describes interventions that use strategies involving naturally–occurring environments and activities, child-responsive interaction styles, and teaching content and strategies derived from developmental science as well as the science of applied behavior analysis. 

In a recent systematic review and meta–analysis of early interventions for children with ASD, Sandbank and colleagues [6] identified a subset of 26 group design studies that examined the effects of NDBIs and found that the NDBIs showed the strongest body of evidence compared to the other intervention types included. However, the NDBI studies had multiple methodological and quality limitations across them, especially where 47.59% used outcome measures that were proximal to the intervention goals and 78.77% measured outcomes in contexts similar to the intervention context. Previous reviews have indicated that studies that use proximal and context-bound measures likely inflate intervention effects [7,8]. Additionally, 47.09% used outcome measures at risk of correlated measurement error (CME) due to the participation of adults in outcome measurement who have been trained in the intervention strategies. Sandbank and colleagues found that the group of 26 NDBIs resulted in significant improvements in social communication, cognition, play, and language, but, when examining results from only those studies that did not rely on a parent report (a measurement type that is susceptible to CME), only play and social communication outcomes showed significant improvements.

### 1.2. Early Start Denver Model

The Early Start Denver Model (ESDM) is an NDBI specifically designed for the needs of very young children with ASD that has been widely studied [9]. The ESDM is one of the few comprehensive early intervention programs for ASD. Although it has a particular focus on autism-specific impairments, it teaches skills across nine developmental domains. The ESDM, which is one of the few commercially available NDBIs, has previously been identified as a promising and cost-effective intervention [10] and has been examined in two systematic reviews. The first review included 15 studies using a variety of study designs [11] and reported overall positive results. However, over half of the included studies had methodological weaknesses. A second review [12] of 10 studies found similar findings and reported that, although most of these studies had positive results, the three comparative studies had mixed findings. Problems of study quality in both meta-analyses included lack of true experimental designs, lack of blind assessment, and small sample sizes. 

The purpose of this meta–analysis was to expand and improve upon the findings of these previous reviews in several ways: by including many more recently published studies, by using a meta-analytic approach that allowed for a quantitative understanding of effects, by focusing on comparative studies, and by examining effects on specific domains as well as overall effects of the intervention. This would help identify strengths and areas needing improvement for a well–known early ASD intervention.

### 1.3. Research Questions

This systematic review and meta-analysis of the effects of the ESDM on outcomes for young children with ASD was conducted to address the following questions: (1) Does the ESDM result in significant improvements in outcomes for young children with ASD, both overall and specifically in the domains of autism symptomology, language, cognition, social communication, adaptive behavior, and repetitive behaviors? (2) Are the findings affected by quality and study design features, including proximity and boundedness of measurement? 

## 2. Materials and Methods

### 2.1. Eligibility Criteria 

Eligibility criteria are presented in Table 1. Studies were included in the meta-analysis if the study enrolled participants with ASD or at risk for ASD under age 6. The intervention type was restricted to the ESDM, but could include individual, group, or parent-implemented ESDM, or interventions that were derived from ESDM (e.g., Infant Start [13]). Study design was restricted to group comparison studies (randomized control trials or quasi experimental designs). Included studies were required to have a non–ESDM treatment comparison group, which could include: treatment as usual, waitlist control, or parent education only, or a treatment comparison that did not include ESDM interventions. Studies that did not have a comparison group (e.g., single case design or pre/post design) were excluded. Studies had to report at least one child outcome that provided adequate information to calculate a standardized mean difference effect size (e.g., means and SDs or F statistics). Studies had to be published in English to be eligible for inclusion due to the language restraints of the coders. Follow-up studies were excluded as the only data from the timepoint closest to the end of the intervention. 

### 2.2. Search Procedure

A total of nine databases were searched through Proquest: (American Psychological Association (APA) PsycArticles, APA PsycInfo, APA PsycTests, Dissertations and Theses at the University of California, Education Resources Information Center (ERIC), Linguistics and Language Behavior Abstracts, PAIS, ProQuest Dissertations and Theses A&I, Sociological Abstracts). The final search was completed in October 2019. Unpublished or “gray” literature was searched using the online databases of dissertations and theses as well as proceedings from relevant conferences (e.g., International Society for Autism Research) and reference lists. The search and study selection process were completed by the first author.

### 2.3. Data Extraction and Coded Variables

All child outcome measures that were reported were recorded from each study. If a study reported both a total or overall score and subscale scores, only the total/overall score was used. However, the subscale scores were used for the appropriate outcome–specific meta–analysis. For example, if a study reported both the overall developmental quotient from the Mullen Scales of Early Learning (MSEL) and the subscales, the overall score was used in the overall outcome analysis, and the expressive and receptive language subscales were used in the language outcomes analysis [14]. Only outcomes from the timepoint most proximal to the end of the intervention were included. 

Study-level characteristics were recorded, including the location in which the study took place, length of intervention delivery (in weeks), intensity of delivery (hours per week), mean child age (in years), percent of participants that were male, the primary person implementing the intervention (parent or professional, which included researcher, teacher, or therapist), whether the intervention included a parent training component, the format of the intervention delivery (individual, group, or mixed), and the fidelity of the intervention implementation, if reported.

Study quality indicators were recorded, including the use of random assignment and the use of assessors who were blind or naïve of the group assignment. The measurement–quality variables were coded using definitions and flowcharts described in Sandbank and colleagues [6]. Each measure was coded according to the proximity and context of the measure.

*Measurement proximity.* Proximity of the measurement was coded as distal or proximal. Distal measures were defined as those behaviors measured using developmentally–scaled tests meant to measure general development. Proximal measures were defined as those in which the measurement directly measured the goals of the intervention. For example, the MSEL would be considered a distal measure, whereas a child’s ability to imitate would be considered a proximal measure since this is a behavior that is specifically targeted in the ESDM curriculum.

*Measurement context.* The context of measurement was coded as generalized or context bound. Generalized outcomes were defined as outcomes that were measured in a context differing from the intervention context of at least one dimension (setting or interaction partner). Context–bound measures (CME) were defined as those that were taken in the same context as the intervention was delivered. For example, measuring a child’s language using a subscale of the MSEL would be coded as generalized because it uses different materials, interaction styles, and a likely interaction between the partner and setting, whereas measuring a child’s language during an intervention session with their usual therapist would receive a context-bound code. Parent questionnaires were coded as generalized because they are intended to capture the child’s generalized tendency to behave in the home context. The use of parent/teacher reports was also coded. Potential for CME was defined as any measure involving an adult trained in the intervention. This included a parent report if and only if the parent had been trained in the intervention.

### 2.4. Analytic Strategies

The standardized mean difference effect size was calculated using Hedges’ g to compare group differences (treatment vs. control) at post-test. Hedges’ *g* corrects the slight bias in Cohen’s *d* that occurs in studies with small sample sizes, and is, therefore, a more conservative estimate of effect in a sample of studies with high variability [15]. When studies did not report means and standard deviations, the effect size was calculated from an F-statistic, derived from a group*time ANOVA to mitigate the concern of effect-size inflation [16]. 

A robust variance estimation (RVE) meta-analysis was conducted using the robumeta package on R [17]. The RVE meta–analysis accounts for the nesting of multiple effect sizes within one study [18]. This method was selected rather than traditional meta-analyses, which use only one effect size per study, to account for the fact that the ESDM targets a variety of skills and its efficacy is generally assessed using more than one outcome measure. Separate meta analyses were conducted for each subskill analysis using separate RVE meta-analyses. Meta-regression analyses were conducted to understand the contributing factors of study-level characteristics (dose and person implementing) and study quality indicators. The heterogeneity of effect sizes was examined using τ^2^ and *I^2^*. Between study variance represented by τ^2^, which is in the metric of the effect size. *I^2^* represents the percent of variability that is true heterogeneity across the observed effect estimates. Higher levels of *I^2^* indicate greater dispersion between effect sizes that may be accounted for with moderator analyses [19]. A *p* < 0.05 alpha level was selected as the level of significance for all analyses.

A primary coder (the first author) read and extracted the data from all studies. A second person independently extracted the data from each study so that all variables on 100% of the included studies were coded by two raters. Overall reliability of independent ratings across all coded measures was 97.2%. Disagreements were resolved by first verifying the information in the manuscript and then by discussing between coders, if needed, until agreement was reached so that 100% agreement on all variables was reached. All statistical analyses were completed using the verified data set. 

Although efforts were made to minimize publication bias by including gray literature searches, analyses were included to detect bias. Publication bias was examined through visual analysis of a funnel plot and the Egger’s test of a small study bias [20].

## 3. Results

### 3.1. Study Selection

**Figure 1:**
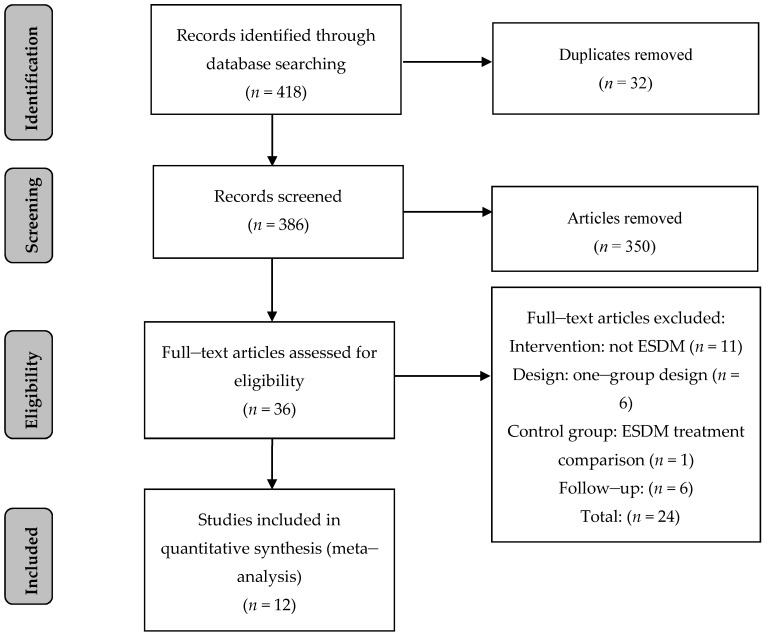
Prisma diagram of study inclusion.

The initial search identified 411 articles to be screened for inclusion. After the initial and full-text screening of the identified articles, 12 studies, including 11 published manuscripts and one dissertation [21], were included in the final analysis. A Preferred Reporting Items for Systematic Reviews and Meta-analyses (PRISMA) flow diagram of exclusion procedures is provided in Figure 1.

### 3.2. Study Characteristics

The 12 included studies were published between 2010 and 2019. The studies took place in five different countries: Australia, Austria, China, Italy, and the United States. The studies included 640 participants (286 intervention and 354 control). The participants ranged in age from nine months to five years old with an average overall age of 2.51 years (SD = 0.89). The studies that reported on gender reported that 80.6% of the samples were male. A total of 44 different effect sizes were reported across the 12 studies. A range of outcome measures were used. Overall study characteristics are shown in Table 2 and characteristics specific to each effect size are shown in Table 3.

In five studies, the parent was the sole agent of implementation. An additional five studies used an intervention approach that incorporated parent coaching but was primarily implemented by a professional. Four studies used a group-based approach: two studies trained parents in groups [21,22] and two studies used group-delivered ESDM [23,24]. Outcomes of studies that included parents did not show significantly higher outcomes than those that did not (B = 0.289, *p* = 0.39). Overall fidelity of implementation was high (mean = 83.2%, range = 75–92%). The studies used a wide range of intervention dosages both in intensity and in length, ranging in intensity from one hour per week to 20 hours per week, and ranging in length from six weeks to 156 weeks. This resulted in total hours of intervention ranging from 12 hours to 2080 hours. However, a meta-regression showed that child outcomes were not significantly related to the length of intervention (B = −0.01, *p* = 0.46), to the hours per week of intervention (B = −0.02, *p* = 0.73), or to the total number of hours (B = 0.004, *p* = 0.66). Additional information about what interventions the control groups received during the study period is included in the Table A1 (Appendix A).

### 3.3. Overall Outcomes

**Figure 2:**
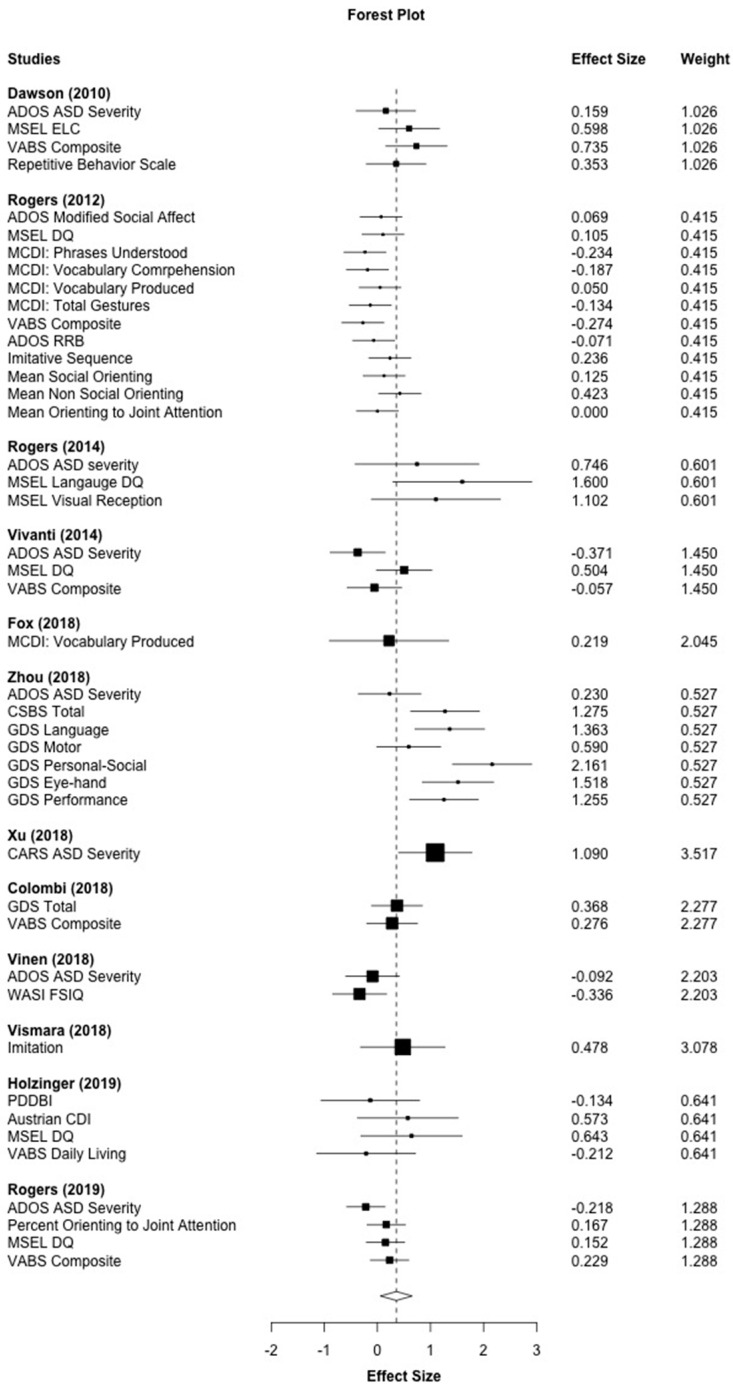
Main effect of the Early Start Denver Model (ESDM) intervention on developmental and symptom outcomes. *Note.* ADOS: Autism Diagnostic Observation Schedule, RRB: Restrictive and Repetitive Behavior, MSEL: Mullen Scales of Early Learning, DQ: Developmental Quotient, ELC: Early Learning Composite, MCDI: MacArthur Bates Communicative Development Inventory, VABS: Vineland Adaptive Behavior Scales, CSBS: Communication and Symbolic Behavior Scales, GDS: Griffith Developmental Scales, CARS: Childhood Autism Rating Scale; WASI FSIQ: Wechsler Abbreviated Scale of Intelligence Full Scale Intelligence Quotient, PDDBI: Pervasive Developmental Disorder Behavior Inventory. *Note.* Black boxes indicate the weight of each effect size and bars indicate the confidence interval. The overall effect size is indicated by the open diamond and dotted line (*g =* 0.357).

Figure 2 shows the results of the RVE meta–analysis examining the effects of the ESDM on all included outcome measures. The effect size weight is shown for each of the 44 outcome measures arranged by the study. Larger black boxes around the effect sizes represent larger weights in the meta–analysis, and bars represent the confidence intervals. The RVE aggregated effect size resulted in an overall effect size of *g* = 0.357 (*p* = 0.024). This moderate and statistically significant effect size suggests a significant advantage for children who received the ESDM intervention compared to children enrolled in control groups. However, a moderate amount of between–study heterogeneity was observed in this analysis (*I*^2^ = 64.84%, τ^2^ = 0.16). The majority of studies showed confidence intervals that overlapped with zero, which indicated that the RVE aggregated effect was driven by a few studies or by specific outcome measures. This further assessed the subgroup analyses below.

### 3.4. Study Quality Indicators

The studies were analyzed for their use of study design elements. Study level quality elements (blind assessors, random assignment) are reported in Table 2, and effect–size specific elements (if the measure was distal, generalized, relied on parent report, or showed potential risk of CME) are reported in Table 3. Thirty-eight of the forty-four (83.2%) elements included measures used developmentally–scaled, distal measures of child outcomes. Forty-three of the included measures (97.7%) used generalized contexts to measure child outcomes. Fourteen outcome measures used parent report measures (31.8%), and 10 outcome measures (22.7%) had potential risk for CME (nine of these 10 studies due to the use of parent report measures). Blind assessors were used in 72.2% of eligible studies (eight out of eleven studies with one study not being included since it only used a parent report so that no assessors were used). Six of the 12 studies (50%) used a randomized study design. A meta regression analysis showed that child outcomes were not significantly associated with distal outcomes (B = 0.28, *p* = 0.47), generalized outcomes (−0.38, *p* = 0.20), parent report (B = −0.08, *p* = 0.70), use of blind assessors (B = 0.15, *p* = 0.74), or the use of a random assignment (−0.02, *p* = 0.95). Furthermore, the inclusion of these variables did not account for the observed heterogeneity (*I*^2^ = 76.22%, *τ*^2^ = 0.26). Because of the high overlap between the use of parent measures and the potential risk of CME, only the variable for the use of parent measures was retained in the meta-regression analysis.

### 3.5. Autism Symptoms

**Figure 3:**
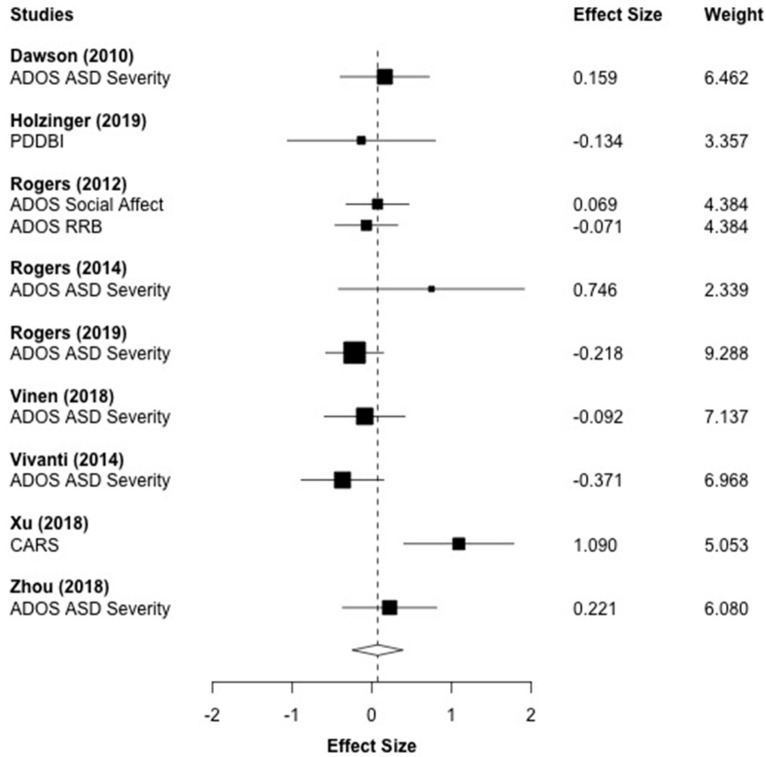
Main effect of the Early Start Denver Model (ESDM) intervention on autism spectrum disorder (ASD) symptomology outcomes. *Note.* ADOS: Autism Diagnostic Observation Schedule, RRB: Restrictive and Repetitive Behavior, CARS: Childhood Autism Rating Scale; PDDBI: Pervasive Developmental Disorder Behavior Inventory.

Figure 3 displays the forest plot for the 10 autism symptomology outcomes that were reported across nine studies. The effect sizes are represented such that positive values indicate a reduction in autism symptomology. The aggregated effect size was *g* = 0.070 (*p* = 0.616), which indicated that children who received ESDM treatment did not show significant improvements in autism symptomology when compared to the control group. A moderate level of heterogeneity was observed (*I*^2^ = 48.90%, *τ*^2^ = 0.073).

### 3.6. Language

**Figure 4:**
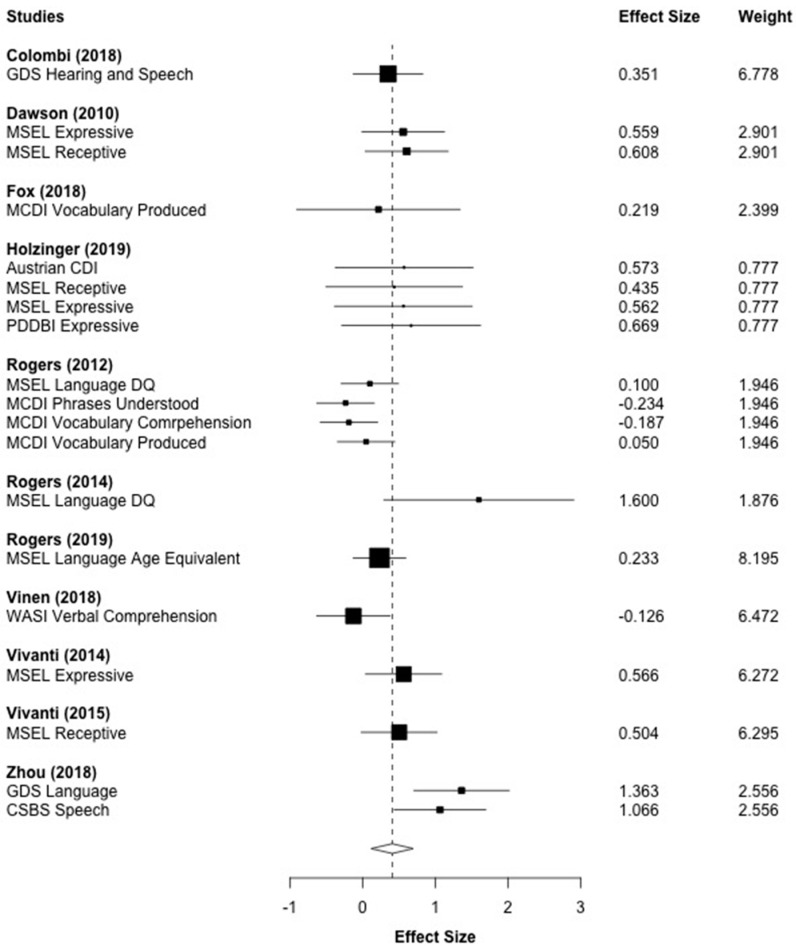
Main effect of ESDM intervention on language outcomes. *Note*. MSEL: Mullen Scales of Early Learning, DQ: Developmental Quotient, MCDI: MacArthur Bates Communicative Development Inventory, GDS: Griffith Developmental Scales, WASI: Wechsler Abbreviated Scale of Intelligence, PDDBI: Pervasive Developmental Disorder Behavior Inventory.

Figure 4 displays the forest plot for the 19 language outcomes that were reported across 11 studies. The effect sizes represent both expressive and receptive language outcomes. The aggregated effect size was *g* = 0.408 (*p* = 0.011), which indicates that children who received the ESDM intervention made significant progress in language development compared to children in the control groups. A moderate level of heterogeneity was observed (*I*^2^ = 52.70%, *τ*^2^ = 0.088).

### 3.7. Cognition

**Figure 5:**
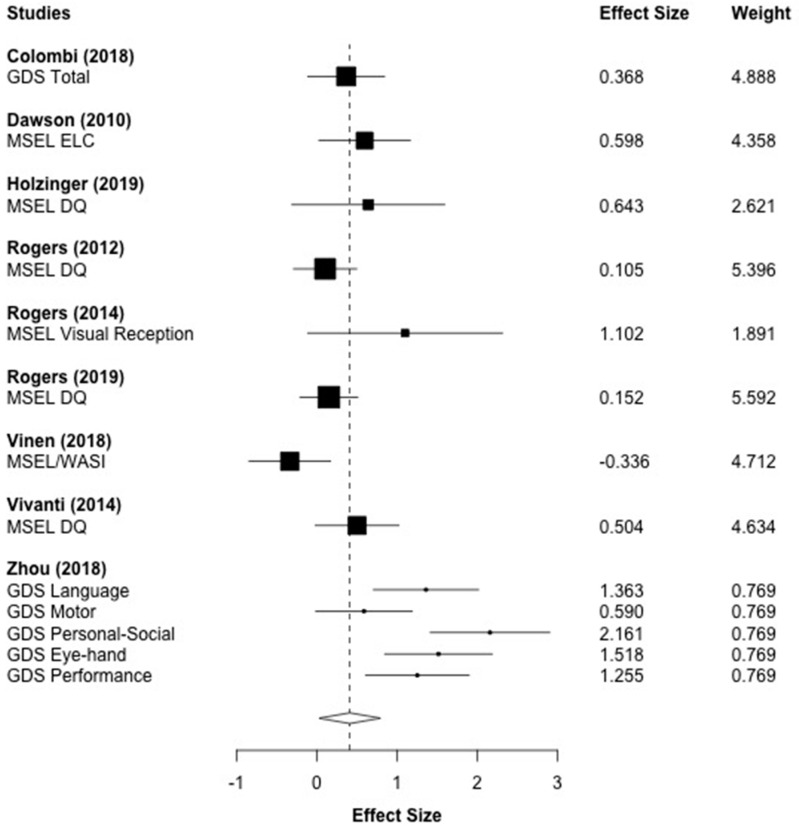
Main effect of ESDM intervention on cognitive outcomes. *Note.* MSEL: Mullen Scales of Early Learning, DQ: Developmental Quotient, ELC: Early Learning Composite, GDS: Griffith Developmental Scales; WASI FSIQ: Wechsler Abbreviated Scale of Intelligence.

Figure 5 displays the forest plot for the 13 cognitive outcomes that were reported across nine studies. The aggregated effect size was *g* = 0.412 (*p* = 0.038), which indicated that children who received the ESDM intervention made significant progress in cognitive development compared to children in the control group. A moderate level of heterogeneity was observed (*I*^2^ = 66.30%, *τ*^2^ = 0.145).

### 3.8. Social Communication

**Figure 6:**
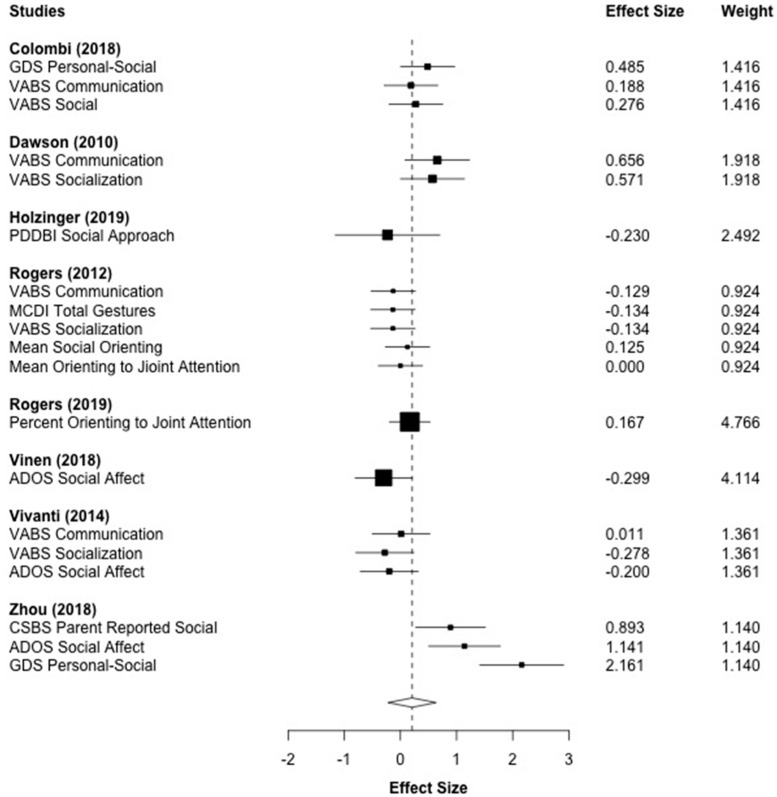
Main effect of ESDM intervention on social communication outcomes. *Note*. ADOS: Autism Diagnostic Observation Schedule, MCDI: MacArthur Bates Communicative Development Inventory, VABS: Vineland Adaptive Behavior Scales, CSBS: Communication and Symbolic Behavior Scales, GDS: Griffith Developmental Scales.

Figure 6 displays the forest plot for the 19 social communication outcomes that were reported across eight studies. This included related sub-scores of the Vineland (Communication and Socialization) [32]. The aggregated effect size was *g* = 0.209 (*p* = 0.285), and was not statistically significant. A high amount of heterogeneity was observed across social communication measures (*I*^2^ = 72.53%, *τ*^2^ = 0.176). 

### 3.9. Adaptive Functioning

**Figure 7:**
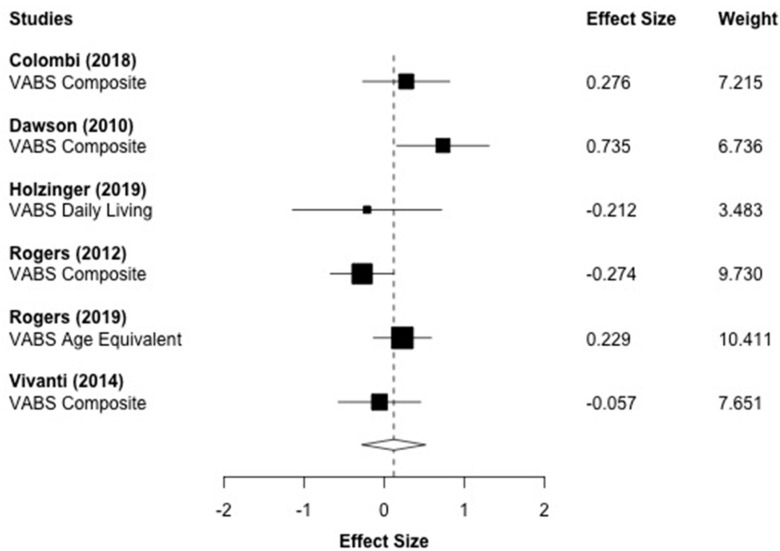
Main effect of ESDM intervention on adaptive functioning outcomes. ***Note.*** VABS: Vineland Adaptive Behavior Scales.

Figure 7 displays the forest plot for the six adaptive functioning outcomes that were reported across six studies. All of the included effect sizes were taken from the Vineland [32]. The aggregated effect size was *g* = 0.121 (*p* = 0.458), which was not statistically significant. A moderate amount of between-study heterogeneity was observed (*I*^2^ = 49.03%, *τ*^2^ = 0.062). 

### 3.10. Repetitive Behaviors

**Figure 8:**
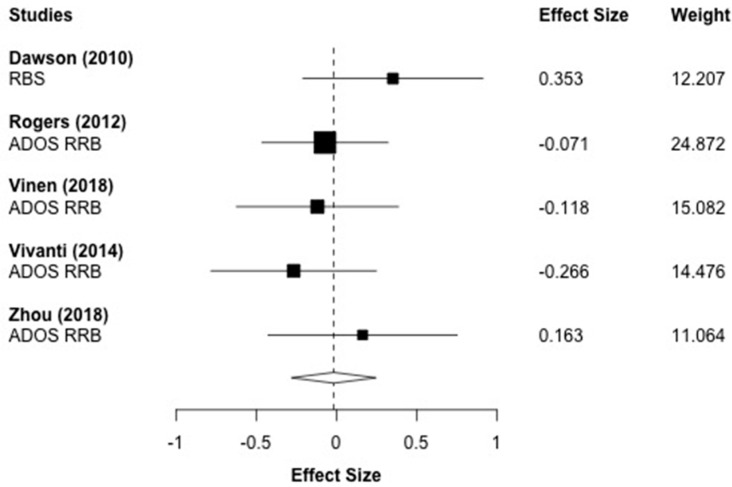
Main effect of ESDM intervention restricted and repetitive behaviors (RRB). *Note*: RBS: Repetitive Behaviors Scale; ADOS RRB: Autism Diagnostic Observation Schedule Restricted and Repetitive Behaviors.

Figure 8 displays the forest plot for the five repetitive behavior outcomes that were reported across five studies. The effect sizes are represented such that positive values indicate a reduction in repetitive behaviors. The aggregated effect size was *g* = −0.016 (*p* = 0.876), which indicated that children who received ESDM treatment did not show significant improvements compared to the control group in repetitive behaviors. This finding should be taken with caution due to the low number of included effect sizes. 

### 3.11. Publication Bias

**Figure A1:**
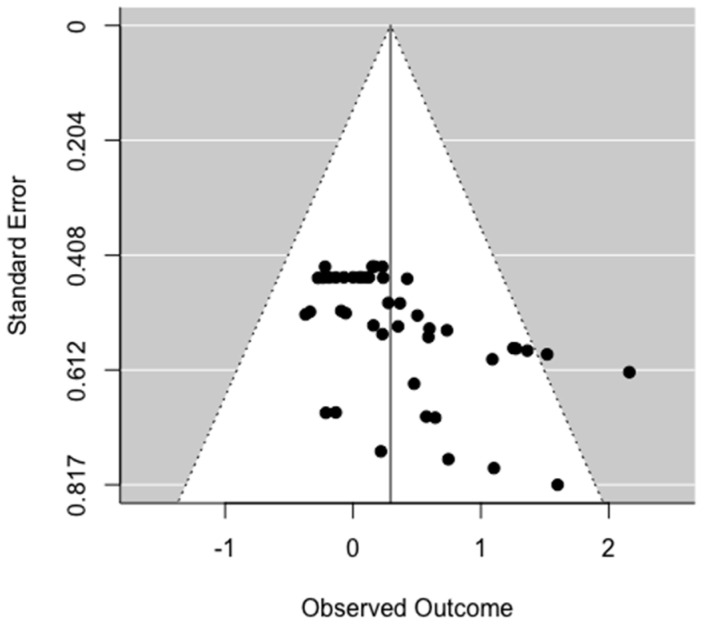
Funnel plot of included studies: effect size and standard error.

An Egger’s test of a small study bias (*p* < 0.01) indicated that there is a risk of a small study bias in this sample. A funnel plot is included in Figure A1 (Appendix B), which shows that two of the 44 effect sizes fall outside of the highlighted area, suggesting a small bias.

## 4. Discussion

This meta-analysis examined the effects of the ESDM for young children with ASD delivered in a variety of formats on a variety of outcomes measures. Across 12 studies that included 44 unique effect sizes, the overall aggregated effect size was *g* = 0.357 (*p* = 0.024). This moderate [33] and statistically significant effect size indicates an overall advantage for children in the ESDM intervention groups compared to children in control groups (*p* = 0.024). (For reference, this represents a gain of 7.84 more points on the Mullen Developmental Quotient than the comparison group.) These significant differences were mostly driven by improvements in cognition (*g =* 0.412) and language (*g =* 0.408). There was a moderate amount of heterogeneity across studies and significant results were not observed for all studies or outcome measures. Nonsignificant differences were observed for the remaining domains: autism symptomology, adaptive behavior, social communication, and restricted and repetitive behaviors (RRBs). Although many of these effect sizes came from one lab, the 12 included studies represent data from five different countries and from interventions of both high and low intensity implemented using a variety of delivery methods including parents, local teachers or therapists, and group-based settings.

One particular strength of this meta-analysis was the general rigor of the measurements used in the included studies. Relatively few measures were at risk of CME, which occurs when measures involve parent interactions with children or parent reports of child skills in studies that have trained parents in the intervention. In the current sample, only 22.7% of studies had potential risk of this source of CME. This is a great deal fewer than the group of NDBI studies that Sandbank and colleagues [6] reported on, which found that 47% of outcomes were at risk for CME. In addition, most studies included in this meta–analysis used norm-referenced measures that were distal (83%) and generalized (97%). This is considerably more than the general pool of NDBI studies included in the Sandbank analysis in which 52% of outcomes used distal measures and 21% used generalized measures. The high rate of distal and generalized measures seen in this current sample of studies reduces concerns of effect size inflation due to the measurement error. 

Given the relative strength in the quality of measurements used in the studies included in the current review, the current findings of significant improvements in language and cognition related to the ESDM compare favorably with previous reviews of ASD interventions. Sandbank and colleagues [6] found that, although NDBIs are generally making significant improvements across domains, the improvements in language and cognitive outcomes as a result of NDBIs were mostly smaller in magnitude (language: *g =* 0.21, *p* < 0.05, cognitive: *g* = 0.18, not significant). In comparison, the present analysis showed significant language and cognitive improvements of *g* = 0.408 and 0.412, respectively. The effect sizes for language in the present ESDM study is also larger than the effect size of *g* = 0.26 reported in a recent meta-analysis that examined language outcomes of multiple types of early ASD interventions [34].

### Limitations and Future Directions

The most prominent limitation was the heterogeneity observed in this sample. This meta-analysis combined a wide range of study designs, measures, and procedures. Twelve of the 44 outcome measures showed results in the negative direction, and the majority of outcomes had a confidence interval that included zero. Thus, the overall positive effect size should be taken cautiously.

Two of the potential contributors to the observed heterogeneity in this analysis involved dosage and delivery. A wide range of dosage was used across the 12 included studies in terms of length of intervention and intensity of intervention. Although neither length or intensity of dosage were significantly related to outcome magnitude, this lack of association should be considered with caution. In terms of delivery, five of the studies used a parent-implemented approach. In these studies, the dosage refers to the amount of time the parent was coached rather than the amount of time the parent used the strategies with the child. Four of the studies used a group–based approach. In this case, intensity of individual receipt of intervention is likely different from studies that used a one–on–one delivery approach. Thus, the true dosage of intervention is hard to quantify in some of these studies. Lack of relationship between dosage and outcomes has also been shown in several previous meta–analyses of early interventions [7,34]. Further study is needed to understand the role of dosage in intervention outcomes. 

A second limitation was in the scientific rigor of the study designs. While all studies were controlled, half of the included studies used a non-randomized control design. Although the meta-regression indicated that there was not a significant relationship between the use of a non-random design and study outcomes, the negative beta weight indicates that randomized controlled studies had smaller effect sizes than the quasi–experimental studies on average. Many of the quasi-experimental studies were carried out outside of university and lab settings, including community implemented studies [28] in which it was not considered feasible or ethical to implement a randomized design. We included these quasi-experimental controlled studies despite the design limitations to represent findings of real-world applications of the ESDM. Other problems with rigor include use of measures based on parent report, outcome measures that were at risk of a correlated measurement error, and non–blinded assessors (in some of the studies). 

A third limitation relates to the subgroup meta-analysis that showed nonsignificant changes on measures of autism symptomology, adaptive behaviors, repetitive behaviors, and social communication. This indicates that the ESDM intervention may be less effective at targeting these characteristics of early ASD. However, in the case of ASD severity and RRBs, this may also be partly due to an issue in measurement. Many of the outcome measures included for these domains came from the Autism Diagnostic Observation Schedule (ADOS) [35]. The ADOS is intended to capture relatively stable characteristics of ASD symptomology including social communication for diagnostic purposes and was not created with the intention of measuring a treatment-related change. A more recent measure, known as the Brief Observation of Social Communication Change (BOSC) [36], was created for this purpose, and may be a more useful tool for capturing change in these outcomes. Future studies should further examine these subdomains using more sensitive outcome measures and should consider additional intervention strategies to specifically target these areas.

A final limitation is the risk of small study bias observed. However, this concern was mitigated by using a correction for small study effects included in the RVE meta-analyses estimation and through extensive searching of gray literature, which included one unpublished study. 

## 5. Conclusions

Based on the moderate and significant overall effect size resulting from this meta-analysis involving 640 participants across 12 studies, the ESDM shows promise as an effective practice for young children with ASD in improving outcomes in some areas affected by early ASD, especially language and cognitive outcomes. Domains involving autism symptomology, social communication, adaptive behaviors, and repetitive behaviors did not show an ESDM advantage and may require additional treatment efforts and/or more sensitive outcome measures. This body of evidence has several strengths in scientific rigor including the use of distal and generalized outcome measures and lowered risk of correlated measurement error compared to other NDBI interventions, but also shows a weakness in the number of quasi-experimental non-randomized study designs. Lastly, the studies reported high fidelity of treatment implementation across a variety of delivery contexts, including five different countries, group and individual settings, and a range of implementors that included parents, community therapists, and teachers.

## Figures and Tables

**Table 1:** Inclusion criteria and search terms.

Inclusion Criteria	Criteria	Corresponding Search Terms
Participants	Autism spectrum disorder, all participants younger than age 6	
Intervention	Early Start Denver Model	“Early Start Denver Model” (Anywhere)
Comparison	Treatment as usual, waitlist control, general information only, referral to other services, or non-ESDM intervention	assign* OR group OR BAU OR “wait list” OR RCT OR random* OR quasi OR control* OR trial (Abstract)
Outcome	Any child outcome	
Study Design	Group design study, including randomized control trial and quasi experimental design	

**Table 2:** Summary descriptions of included studies.

Author (year) [ref]	Country	Participants (*n*)	Average Age (Years)	Percent Male	Intervention Length (Weeks)	Hours per Week	Primary Implementer	Parent Coaching	Group	Fidelity	Blind Assessors	Random Assignment
Dawson (2010) [25]	USA	48	1.95	72	104	20	Professional	yes	no	85%	yes	yes
Rogers (2012) [26]	USA	98	1.75	77.55	12	1	Parent	yes	no	75%	yes	yes
Rogers (2014) [13]	USA	11	0.75	65.63	18	1	Parent	yes	no	91%	yes	no
Vivanti (2014) [23]	Australia	57	3.4	87.72	52	15	Professional	yes	yes	92%	no	no
Fox (2018) [21]	USA	10	2.73	70	6	3	Parent	yes	yes		NA	yes
Zhou (2018) [22]	China	43	2.21	88.37	26	1.5	Parent	yes	mix	80%	yes	no
Xu (2018) [27]	China	40	3.77	88.75	8	5	Professional	yes	no	85%	yes	yes
Colombi (2018) [28]	Italy	92	2.76		24	6	Professional	no	no	80% *	no	no
Vinen (2018) [24]	USA	59	3.11	88.1	156 ^†^	15–20	Professional	yes	yes	80% *	yes	no
Vismara (2018) [29]	USA	30	2.46	70.83	12	1.5	Parent	yes	no		yes	yes
Holzinger (2019) [30]	Austria	16	3.62	100	52	4.6	Professional	no	no	80% *	no	no
Rogers (2019) [31]	USA	118	1.72	77.97	116	16	Professional	yes	no	84%	yes	yes

*Note.*^†^ Indicates an average duration. * Indicates a minimum level of fidelity.

**Table 3:** Summary descriptions of included effect sizes.

Study Author (Year) [ref]	Outcome Measure	Post-Test Mean (SD): Intervention Group	Post-Test Mean (SD): Control Group	Hedges’ *g* (SE)	Distal	General-ized	Parent Report	CME
Dawson (2010) [25]	ADOS ASD severity	7 (1.9)	7.3 (1.8)	0.16 (0.28)	Yes	Yes	No	No
	MSEL ELC	78.6 (24.2)	66.3 (15.3)	0.60 (0.29)	Yes	Yes	No	No
	VABS Composite	68.7 (15.9)	59.1 (8.8)	0.73 (0.29)	Yes	Yes	Yes	Yes
	Repetitive Behavior Scale	16.7 (13.1)	22.0 (16.3)	0.35 (0.29)	Yes	Yes	Yes	Yes
Rogers (2012) [26]	ADOS Modified Social Affect	26.6 (10.1)	27.3 (10.6)	0.07 (0.20)	Yes	Yes	No	No
	MSEL DQ	69.8 (17.9)	67.9 (17.9)	0.11 (0.20)	Yes	Yes	No	No
	MCDI: Phrases Understood	12.7 (9.11)	14.8 (8.1)	−0.23 (0.20)	Yes	Yes	Yes	Yes
	MCDI: Vocabulary Comprehension	106.5 (96.8)	125.7 (106.4)	−0.19 (0.20)	Yes	Yes	Yes	Yes
	MCDI: Vocabulary Produced	42.3 (62.0)	38.9 (73.7)	0.05 (0.20)	Yes	Yes	Yes	Yes
	MCDI: Total Gestures	28.02 (12.6)	29.8 (13.5)	−0.13 (0.20)	Yes	Yes	Yes	Yes
	VABS Composite	77.4 (9.6)	80.3 (11.3)	−0.27 (0.20)	Yes	Yes	Yes	Yes
	ADOS RRB	4.0 (1.9)	3.8 (2.0)	−0.07 (0.20)	Yes	Yes	No	No
	Imitative Sequence	4.6 (3.5)	3.8 (3.4)	0.24 (0.20)	No	Yes	No	No
	Mean Social Orienting	0.5 (0.3)	0.4 (0.4)	0.13 (0.20)	No	Yes	No	No
	Mean Non-Social Orienting	0.7 (0.3)	0.6 (0.4)	0.42 (0.20)	No	Yes	No	No
	Mean orient to Joint Attention	0.3 (0.3)	0.3 (0.3)	0.00 (0.20)	No	Yes	No	No
Rogers (2014) [13]	ADOS ASD severity	3.3 (3.4)	6.3 (3.9)	0.75 (0.59)	Yes	Yes	No	No
	MSEL Language	92.4 (29.5)	45.6 (20.3)	1.60 (0.67)	Yes	Yes	No	No
	MSEL Visual reception	96.1 (16.4)	78.7 (9.3)	1.10 (0.62)	Yes	Yes	No	No
Vivanti (2014) [23]	ADOS ASD Severity	6.9 (2.3)	6.1 (1.6)	−0.37 (0.26)	Yes	Yes	No	No
	MSEL DQ	67.2 (20.2)	56.3 (22.5)	0.50 (0.27)	Yes	Yes	No	No
	VABS Composite	72.1 (13.5)	73.0 (15.5)	−0.06 (0.26)	Yes	Yes	Yes	Yes
Fox (2018) [21]	MCDI: Vocabulary Produced	164.4 (188.2)	124.2 (140.7)	0.22 (0.57)	Yes	Yes	Yes	Yes
Zhou (2018) [22]	ADOS ASD Severity	6.3 (1.3)	6.6 (1.1)	0.23 (0.30)	Yes	Yes	No	No
	CSBS Total	40.9 (8.1)	29.1 (10.3)	1.27 (0.33)	Yes	Yes	Yes	Yes
	GDS Language	68.6 (22.1)	37.7 (22.5)	1.36 (0.33)	Yes	Yes	No	No
	GDS Motor	80.3 (15.1)	71.7 (13.3)	0.59 (0.31)	Yes	Yes	No	No
	GDS Personal-Social	74.5 (10.6)	51.0 (10.8)	2.16 (0.38)	Yes	Yes	No	No
	GDS Eye-hand	76.0 (13.3)	56.1 (12.4)	1.52 (0.34)	Yes	Yes	No	No
	GDS Performance	75.2 (10.3)	58.9 (15.1)	1.25 (0.33)	Yes	Yes	No	No
Xu (2018) [27]	ASD CARS severity	30.4 (5.5)	37.3 (6.7)	1.09 (0.35)	Yes	Yes	No	No
Colombi (2018) [28]	GDS Total			0.37 (0.24)	Yes	Yes	No	No
	VABS Composite			0.28 (0.24)	Yes	Yes	Yes	No
Vinen (2018) [24]	ADOS ASD Severity	8.0 (2.6)	7.8 (2.1)	−0.09 (0.26)	Yes	Yes	No	No
	WASI FSIQ	76.1 (20.8)	82.8 (18.5)	−0.34 (0.24)	Yes	Yes	No	No
Vismara (2018) [29]	Imitation	1.4 (1.0)	0.9 (0.8)	0.48 (0.41)	No	No	Yes	Yes
Holzinger (2019) [30]	PDDBI	41.7 (13.1)	40.0 (10.7)	−0.13 (0.47)	Yes	Yes	Yes	No
	Austrian CDI	324.7 (201.9)	193.8 (229.5)	0.57 (0.48)	Yes	Yes	Yes	No
	MSEL DQ	63.5 (20.2)	50.0 (19.5)	0.64 (0.49)	Yes	Yes	No	No
	VABS Daily Living	81.9 (17.4)	85.4 (13.5)	−0.21 (0.47)	Yes	Yes	Yes	No
Rogers (2019) [31]	ADOS ASD severity	6.7 (2.0)	6.2 (2.5)	−0.22 (0.18)	Yes	Yes	No	No
	Response to joint attention	76.1 (26.9)	70.7 (36.6)	0.17 (0.18)	No	Yes	No	No
	MSEL DQ	83.1 (26.1)	79.1 (25.6)	0.15 (0.18)	Yes	Yes	No	No
	VABS Composite	39.8 (12.1)	36.7 (14.3)	0.23 (0.18)	Yes	Yes	Yes	Yes

*Note.* ADOS: Autism Diagnostic Observation Schedule, RRB: Restrictive and Repetitive Behavior, MSEL: Mullen Scales of Early Learning, DQ: Developmental Quotient, ELC: Early Learning Composite, MCDI: MacArthur Bates Communicative Development Inventory, VABS: Vineland Adaptive Behavior Scales, CSBS: Communication and Symbolic Behavior Scales, GDS: Griffith Developmental Scales, CARS: Childhood Autism Rating Scale; WASI FSIQ: Wechsler Abbreviated Scale of Intelligence Full Scale Intelligence Quotient, PDDBI: Pervasive Developmental Disorder Behavior Inventory, CME: Correlated Measurement Error.

## Appendix A

**Table A1:** Description of the control group.

Author (Year)	Intervention Group (*n*)	Control Group (*n*)	Control Group
Dawson (2010) [25]	24	24	Treatment as usual, plus intervention recommendations, community referrals, and reading material
Rogers (2012) [26]	49	49	Treatment as usual
Rogers (2014) [13]	7	25	Treatment as usual
Vivanti (2014) [23]	27	30	Group-based “generic” intervention program for children with autism spectrum disorders (ASD)
Fox (2018) [21]	5	5	Waitlist
Zhou (2018) [22]	23	20	Treatment as usual
Xu (2018) [27]	20	20	Eclectic intervention services matching the amount of time the Early Start Denver Model (ESDM) group received
Colombi (2018) [28]	22	70	Treatment as usual
Vinen (2018) [24]	31	28	Group-based eclectic intervention program for children with ASD
Vismara (2018) [29]	16	14	Monthly check-ins and access to online material
Holzinger (2019) [30]	7	6	Treatment as usual
Rogers (2019) [31]	55	63	Treatment as usual
